# Forager and farmer evolutionary adaptations to malaria evidenced by 7000 years of thalassemia in Southeast Asia

**DOI:** 10.1038/s41598-021-83978-4

**Published:** 2021-03-11

**Authors:** Melandri Vlok, Hallie R. Buckley, Justyna J. Miszkiewicz, Meg M. Walker, Kate Domett, Anna Willis, Hiep H. Trinh, Tran T. Minh, Mai Huong T. Nguyen, Lan Cuong Nguyen, Hirofumi Matsumura, Tianyi Wang, Huu T. Nghia, Marc F. Oxenham

**Affiliations:** 1 Department of Anatomy, University of Otago, Dunedin, New Zealand; 2 School of Archaeology and Anthropology, The Australian National University, Canberra, Australia; 3 College of Medicine and Dentistry, James Cook University, Townsville, Australia; 4 Institute of Archaeology, Hanoi, Vietnam; 5 School of Health Sciences, Sapporo Medical University, Sapporo, Japan; 6 Department of Archaeology, University of Cambridge, Cambridge, UK; 7 College of Arts, Society & Education, James Cook University, Townsville, Australia

**Keywords:** Archaeology, Biological anthropology, Anaemia

## Abstract

Thalassemias are inherited blood disorders that are found in high prevalences in the Mediterranean, Southeast Asia and the Pacific. These diseases provide varying levels of resistance to malaria and are proposed to have emerged as an adaptive response to malaria in these regions. The transition to agriculture in the Holocene has been suggested to have influenced the selection for thalassemia in the Mediterranean as land clearance for farming encouraged interaction between *Anopheles* mosquitos, the vectors for malaria, and human groups. Here we document macroscopic and microscopic skeletal evidence for the presence of thalassemia in both hunter-gatherer (Con Co Ngua) and early agricultural (Man Bac) populations in northern Vietnam. Firstly, our findings demonstrate that thalassemia emerged prior to the transition to agriculture in Mainland Southeast Asia, from at least the early seventh millennium BP, contradicting a long-held assumption that agriculture was the main driver for an increase in malaria in Southeast Asia. Secondly, we describe evidence for significant malarial burden in the region during early agriculture. We argue that the introduction of farming into the region was not the initial driver of the selection for thalassemia, as it may have been in other regions of the world.

## Introduction

Thalassemias, a group of inherited hemoglobin blood disorders^1^, are highly prevalent in present day Southeast Asia and the Pacific. These diseases stand testament to a deep history of genetic adaptation to the parasitic disease malaria^2,3^. However, it is unknown when thalassemias or malaria originated in the region. The two forms of the disease, alpha and beta thalassemia, cause disruptions to the synthesis of the alpha or beta hemoglobin chains respectively. Malformation of hemoglobin results in an excess of the opposing hemoglobin chain which is the cause of disease in the body^4,5^. Today, malaria afflicts approximately 8 million people in Southeast Asia. Eradication efforts are sporadic and reliant on access to treatment at a community level to be effective^6^. Thalassemias, like other hemoglobin disorders, disrupt the mechanism for malarial parasite-binding to red blood cells^2^. In Southeast Asia where thalassemia genes are observed in high frequencies, in as much as over 75% of the population, malaria is also highly endemic, particularly the most lethal form *Plasmodium falciparum*^3^.

Although most variants of alpha thalassemia provide resistance to malaria with little clinical complication, most homozygous beta thalassemia variants (thalassemia major and intermedia) can have significant effects on health. Complications of beta thalassemia include gross changes to the skeleton and death from infection or iron overload^3,7,8^. In Southeast Asia specifically, a milder form of beta thalassemia, the hemoglobin-E (HbE) variant, is also present in high frequencies, with the gene present in up to 30–50% of the population in some geographical areas^9^. Homozygous HbE is not associated with clinical symptoms. However, complications can occur with co-inheritance of HbE and classical beta thalassemia variants (HbE beta thalassemia)^7,10^. HbE beta and classic beta thalassemias are frequently found co-inherited with alpha thalassemia in Southeast Asia and result in a wide spectrum of symptoms and severity^11^. Modern day research demonstrates that variants within Southeast Asia and the Pacific can vary across short distances, and relate to the ecological ranges of *Anopheles* mosquitos, the vectors for malarial parasites^3^. Given the co-evolutionary relationship that continues to drive high frequencies of thalassemia in the Southeast Asian region today, it can be hypothesized that the identification of thalassemia in the prehistoric record can be, at least in part, related to a prior selection pressure of malaria.

**Figure 1:**
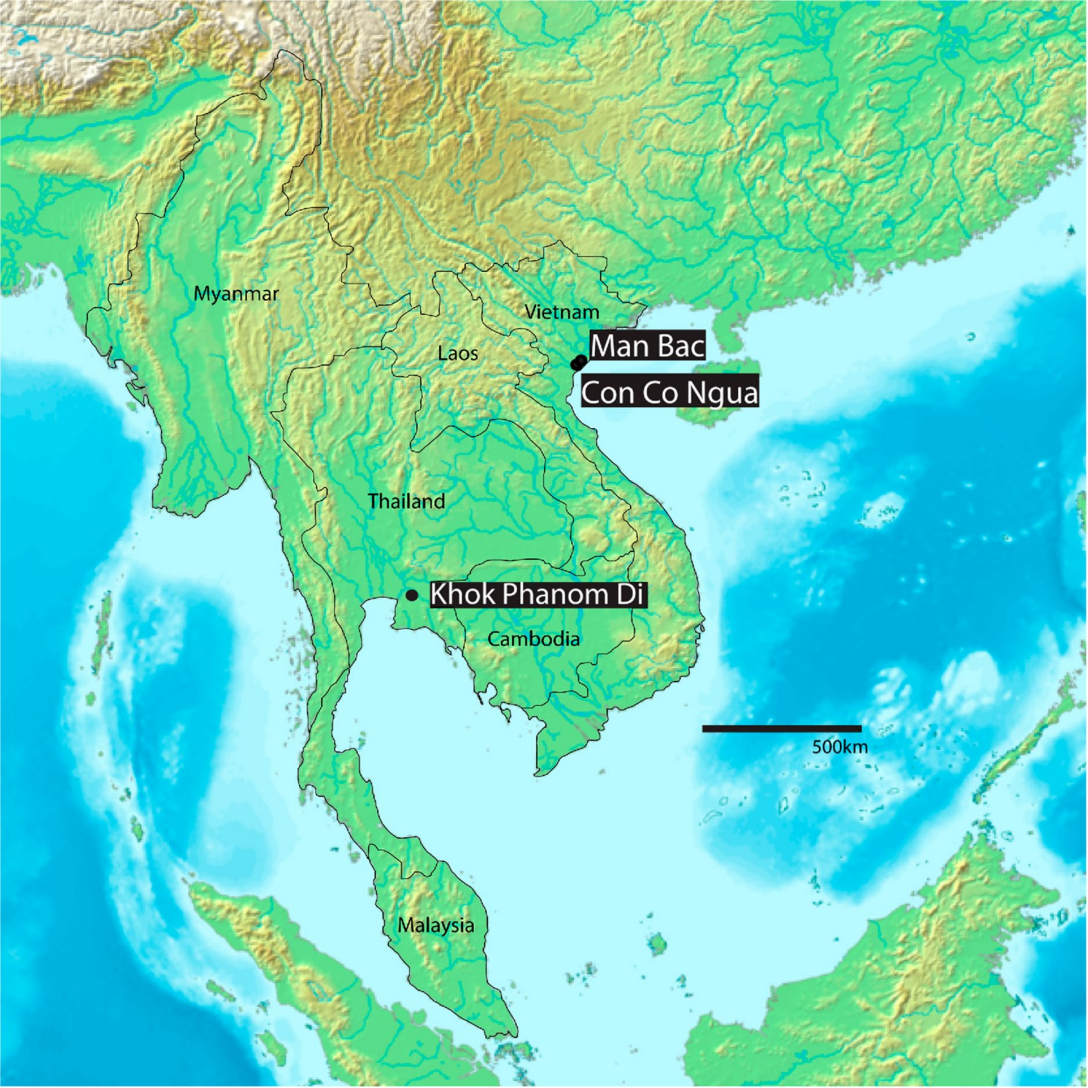
Map of Southeast Asia with sites important for this study. Khok Phanom Di in Thailand provides the only prehistoric evidence of thalassemia in the region. Our study includes research from Man Bac and Con Co Ngua in northern Vietnam. Modified from image by Koba-chan (https://upload.wikimedia.org/wikipedia/commons/1/15/Topographic30deg_N0E90.png) created from DEMIS Mapserver (http://www2.demis.nl/worldmap/mapper.asp). Published under CC BY-SA 3.0 (https://creativecommons.org/licenses/by-sa/3.0/deed.en).

To date, the only archaeological evidence for hereditary anemia in the region dates from approximately 4000–3500 BP from Khok Phanom Di, a Neolithic site in central Thailand (Fig. 1)^12^. In the Mediterranean region, where beta thalassemia is similarly frequent, skeletal evidence in prehistoric assemblages supports the emergence of the disease with the transition to agriculture (the Neolithic) from approximately 7000 years ago^13^. It is hypothesized that agricultural practices such as land clearance encouraged contact between human groups and *Anopheles* mosquitos^13,14^. Did these conditions then emerge alongside the spread of agriculture in Southeast Asia as is proposed to be the case in the Mediterranean?

The emergence of farming in Mainland Southeast Asia (MSEA) occurred much later than the Mediterranean, from only after 4500 years ago^15^, with most Neolithic sites post-dating 4,000 BP. Prior to this time the region was occupied by indigenous Pre-Neolithic foragers descended from the first people out of Africa and into Asia^16^. By the early seventh millennium BP some forager groups in northern Vietnam and southern China developed large sedentary settlements, at the same time as agriculture was practiced to the north in China^17^. The subsequent adoption of agriculture in MSEA during the Neolithic (4500–3100 BP) was associated with multiple migration events of farmers from what is now geo-politically southern China, coming into contact with these forager groups^15,18,19^. These subsistence transition and migration events significantly altered the demography and genetics of this region from this point forward. The aim of this research is to investigate, for the first time, whether thalassemia, as a proxy for malaria burden, was present prior to agriculture in northern Vietnam, and to what degree the transition to agriculture may have contributed to the emergence of thalassemia in Southeast Asia. Additionally, if thalassemia is observed we aim to further investigate which variants may have been present.

We applied a diagnostic protocol based on macroscopically observed dry bone lesions for thalassemia to two archaeological human skeletal assemblages. The Pre-Neolithic site of Con Co Ngua radiocarbon dated to at minimum 6200–6700 cal BP^17,18^ represented a pre-agricultural but sedentary forager community (n = 155; Fig. 1). The Man Bac site dating to 3,906–3,523 cal BP was occupied during the agricultural transition of Southeast Asia (n = 70). This site captures co-habitation of indigenous forager and migrant farmers during the early stages of the agricultural transition^18^. To support the strength of the disease diagnosis using the macroscopic methods, we also conducted histological analysis on three Con Co Ngua individuals to assess microscopic pathological changes in affected bone.

## Results

### Macroscopic results present strong evidence for thalassemia at Man Bac

**Figure 2:**
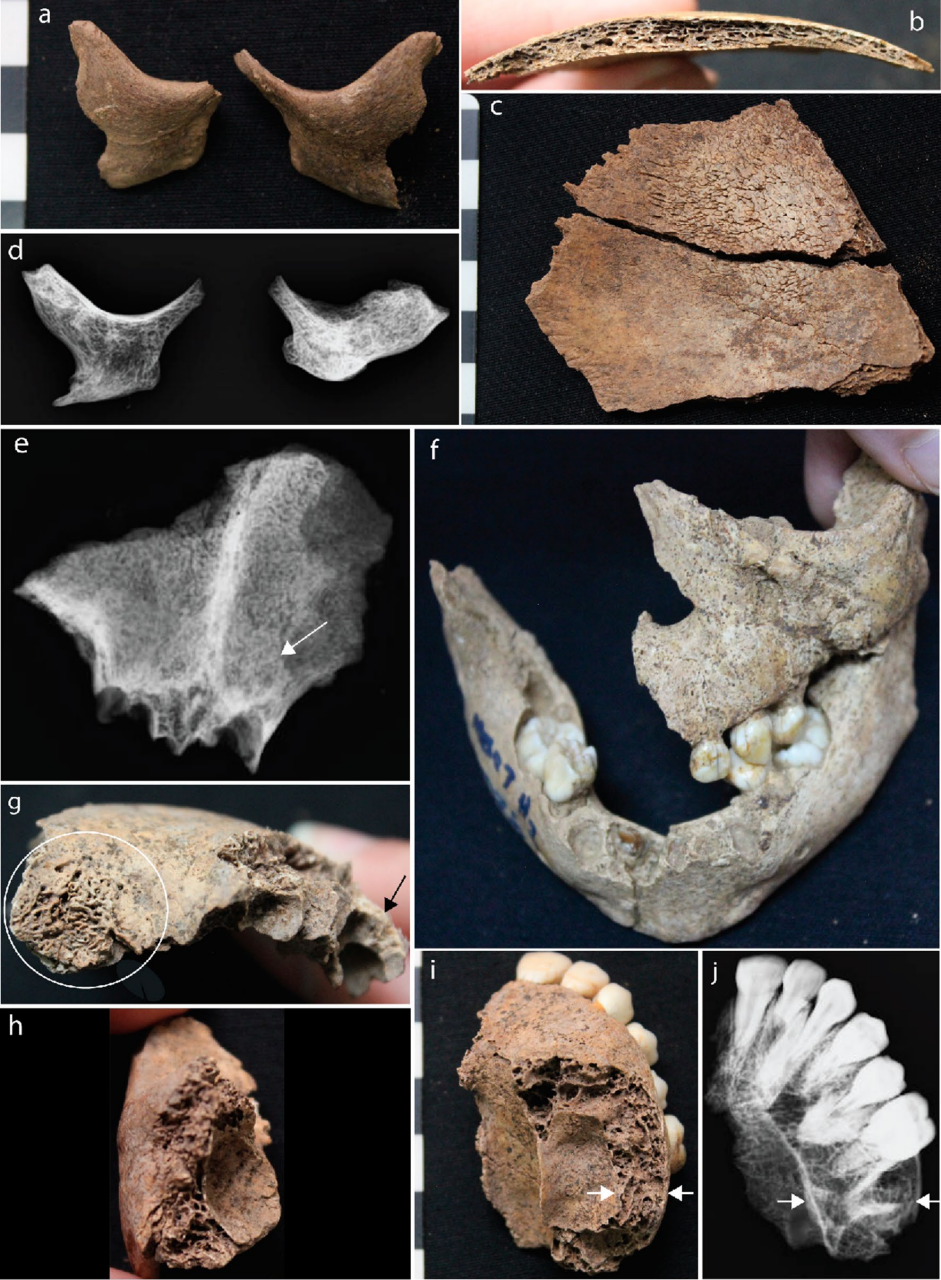
Cranial evidence for thalassemia at Man Bac. (**a**) Anterior protrusion of the zygomatic bones consistent with rodent facies (MB05M3, approx. 6 months old, antero-lateral aspect). (**b**) and (**c**) Diploic expansion of the cranial vault. There is no porosity on the ectocranium but hair-on-end formations are present on the endocranium (MB05M12, approx. 2 years, lateral aspect). (**d**) Marrow hyperplasia of the zygomatic bones (MB05M12, antero-posterior view). (**e**) Lack of pneumatization of the frontal sinus (MB07H1M1, approx. 12 years, antero-posterior view). (**f**) Rodent facies of the maxilla, mandible and zygoma (MB07H2M26, approx. 1.5 years, antero-superior aspect). (**g**) Severe cribra orbitalia (white circle) and diploic expansion of the crania (black arrow, antero-lateral aspect) (MB07H1M1). (**h**) Marrow hyperplasia of the left zygoma (MB07H1M1, lateral aspect). (**i**) and (**j**) Marrow hyperplasia of the maxilla (MB07H1M1, superior-inferior view). The expanse of the marrow hyperplasia is indicated by the white arrows.

**Figure 3:**
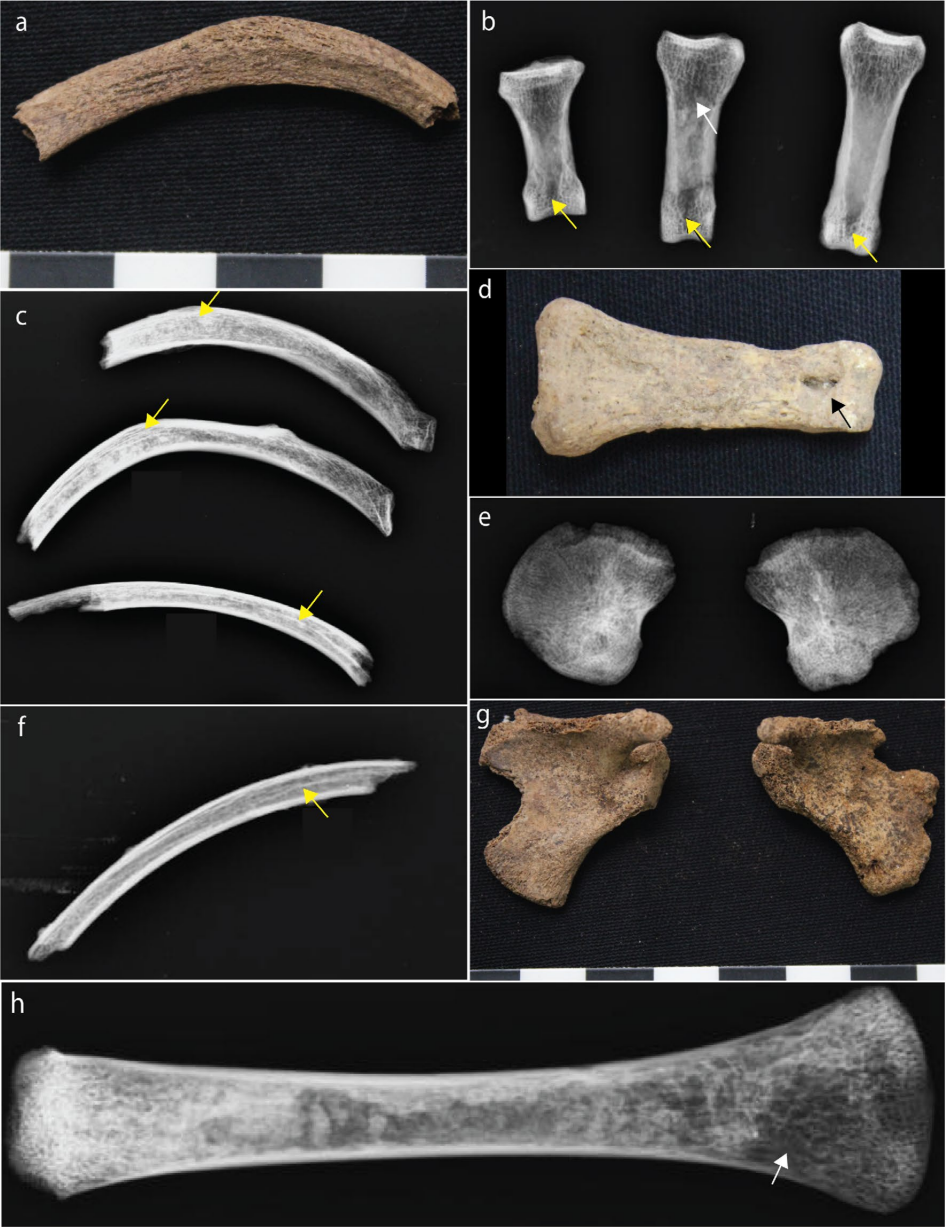
Postcranial evidence for thalassemia at Man Bac. (**a**) Enlarged rib (MB05M3, approx. 6 months old, superior aspect). (**b**) and (**d**) Expanded foramina of the phalanges (yellow arrows) with marrow hyperplasia (white arrow, MB07H1M8, middle aged adult, antero-posterior view). (**c**) “Rib-within-a-rib” sign (yellow arrows, MB07H1M8, supero-inferior view). (**e**) Alteration of the trabecular structure of the ilia. Note the radiating pattern (MB07H1M12, neonate, antero-posterior view). (**f**) “Rib-within-a-rib” sign (yellow arrow, MB07H2M26, approx. 1.5 years, supero-inferior view). (**g**) Enlargement of the scapular spines (MB07H1M12, neonate, posterior aspect). (**h**) Marrow hyperplasia of the humerus (white arrow, MB05M12, approx. 2 years, antero-posterior view).

Five children aged between 6 months and 12 years and one adult presented with skeletal changes that are strongly diagnostic (pathognomonic) for thalassemia (Table 1, Supplementary Text S1; Supplementary Table S1; Figs. 2, 3). These changes included rodent facies, a skeletal condition specific to thalassemia, where expansion of the marrow results in a bulbous face and mandible. Radiographs confirm marrow expansion, a clinical consequence of severe thalassemia (Fig. 2). Radiographic ‘rib-within-a-rib sign’, again pathognomonic for thalassemia, was identified in three individuals. This condition results from extensive marrow expansion within the shafts of the ribs. Further evidence for thalassemia included a lack of the development of facial and cranial sinuses, commonly caused by extensive marrow expansion of the face since infancy. Two children also presented with severe porosity of the orbits and the endocranium, likely due to marrow expansion through the cortical margins of the skull, as a result of thalassemia. Additionally, a newborn with multiple lesions suggestive of possible thalassemia, including evidence of marrow expansion throughout the skeleton was identified (Table 1, Figs. 2, 3).

**Table 1:** Macroscopic and radiographic diagnosis of thalassemia in Man Bac individuals.

Individual ID	MB07H1M8	MB07H1M12*	MB07H2M26	MB05M12	MB05M3	MB07H1M1
Age (years)	30–39	0	1.5	2	0.5	12
Sex	Male	N/A	N/A	N/A	N/A	N/A
**Diagnostic criteria**						
Marrow hyperplasia of the facial bones: maxillae-leading to ventral displacement of central incisors, zygomatic bones-leading to orbital displacement, and/or mandible (rodent facies deformity) (SD)	A	N/A	P	P	P	P
Radiographic: “rib-within-a-rib” appearance. Radiographically defined sclerotic bands within the ribs due to extramedullary hematopoiesis (SD)	**P**	A	P		P	A
Poor or lack of pneumatization of the paranasal and cranial sinuses sparing the ethmoid sinuses (D)	A	N/A	P	A**	A	P
Enlarged tubular bones of the hands and feet due to marrow hyperplasia (infants) sometimes associated with enlarged nutrient foramina *or* Radiographic: coarse trabecular patterns of the hands or feet, sometimes associated with cyst-like lucencies due to focal collection of hyperplasic marrow (D)	P	N/A	N/A	N/A	A	N/A
Premature fusion of epiphyseal plates particularly of the proximal humerus and distal femur, often causing short long bone maximum length (D)	A	A	A	A	A	N/A
Widening of entire rib, or widening of the rib head and neck with pronounced bulbous appearance posteriorly (costal osteomas). Associated with radiograph appearance of erosion of the inner cortex (D)	P	A	A	A	P	A
Diagnosis	Probable	Possible	Probable	Probable	Probable	Probable

P = present, A = absent, N/A = skeletal element missing/unobservable.

*MB07H1M12 presented with multiple suggestive lesions, but no diagnostic (D) or strongly diagnostic (SD) lesions (see Supplementary Table S1).

**Frontal sinuses were not preserved, absence of pneumatization based on preservation of maxillary sinuses only.

### Macroscopic results present evidence suggestive but not diagnostic for thalassemia at Con Co Ngua

We identified seven adults and adolescents at Con Co Ngua presenting with macroscopic and radiographic ‘bone-within-a-bone’ changes of the limb bones (Supplementary Fig. S1; Supplementary Table S1). We observed a mixture of enlargement and restriction of the medullary canal areas in these individuals. In thalassemia, this skeletal change is caused by marrow expansion within the long bones perforating the outer cortex. As such, these pathologies are suggestive of thalassemia but alone are not diagnostic for thalassemia, as they can also be found in a number of other chronic conditions^20^ (Supplementary text S1). However, these skeletal changes were similar to those with strong diagnostic evidence of thalassemia at Man Bac. We extracted bone samples from three of the CCN individuals for further microscopic analysis.

### Combined microscopic and macroscopic results strongly suggest thalassemia at Con Co Ngua

**Figure 4:**
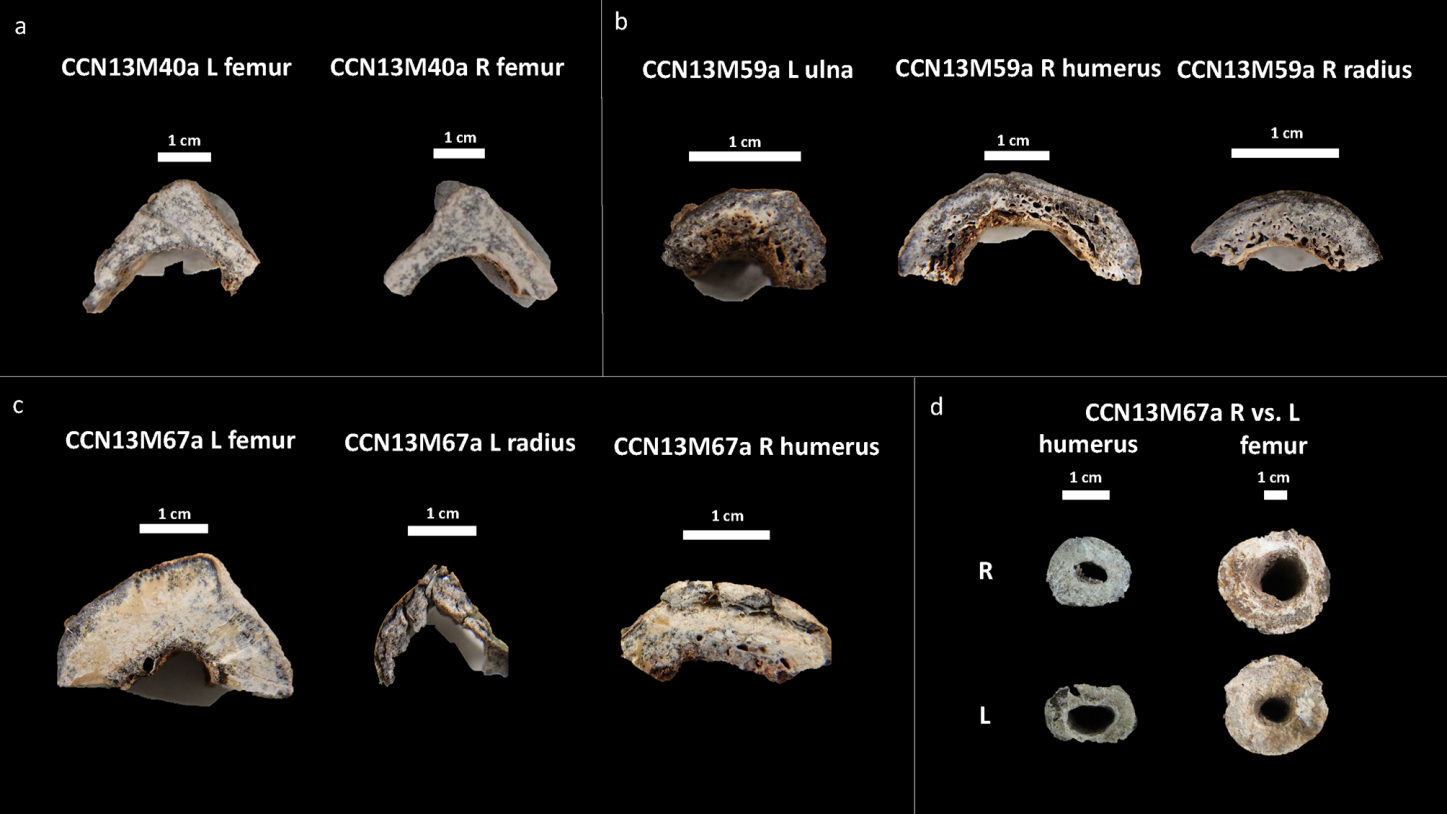
Cross section of microscopic samples from Con Co Ngua. (**a**) Femora sections from CCN13M40a. There is increased cortical thickness, and the cortical width of right femur is asymmetrically wider than the left. (**b**) Upper limb sections from CCN13M40a. Large endocortical pores are evident. Medullary canal widening is most distinct on the humerus. (**c**) Upper and lower limb sections from CCN13M67a. While the femur presents with extreme cortical thickness and restriction of the medullary canal area, the upper limb sections present with severe porosity of the endocortical surfaces. (**d**) Asymmetry of the medullary canals of the humeri and femora of CCN13M67a.

**Figure 5:**
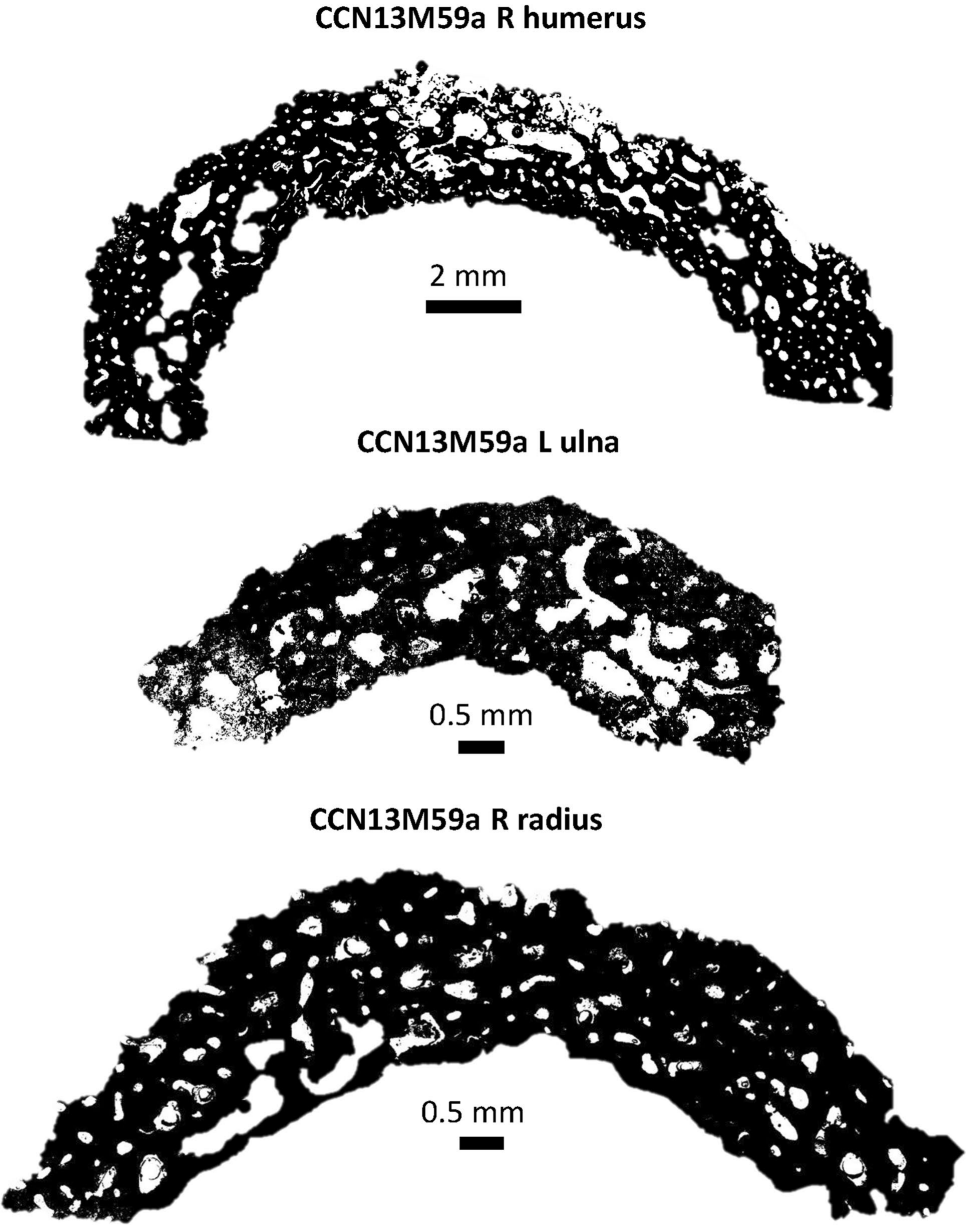
Macroporosity observed in the arm bones of CCN13M59a. The porosity is large and coalescing.

We assessed bone samples of one adolescent (> 16 years) of unknown sex (CCN13M67a), and two adults: CCN13M40a (young adult female), CCN13M59a (old adult male) (Fig. 4). We focused specifically on the endocortex (cortical bone located on the inner third portion of the cross-section) as preliminary macroscopic assessment of the samples prior to microscopy determined this region to be particularly important (Fig. 4). Widespread pores were evident on the endocortical surfaces of samples from the old adult male (CCN13M59a). The porosity consists of ‘giant’ pores that appear to have been created as a result of adjacent coalescing pores (Figs. 4, 5). All three bones of this individual showed a “trabecularization effect”, which essentially transformed cortical bone into a trabeculae-like matrix of bone as a result of prolonged resorption, and possibly marrow expansion.

**Figure 6:**
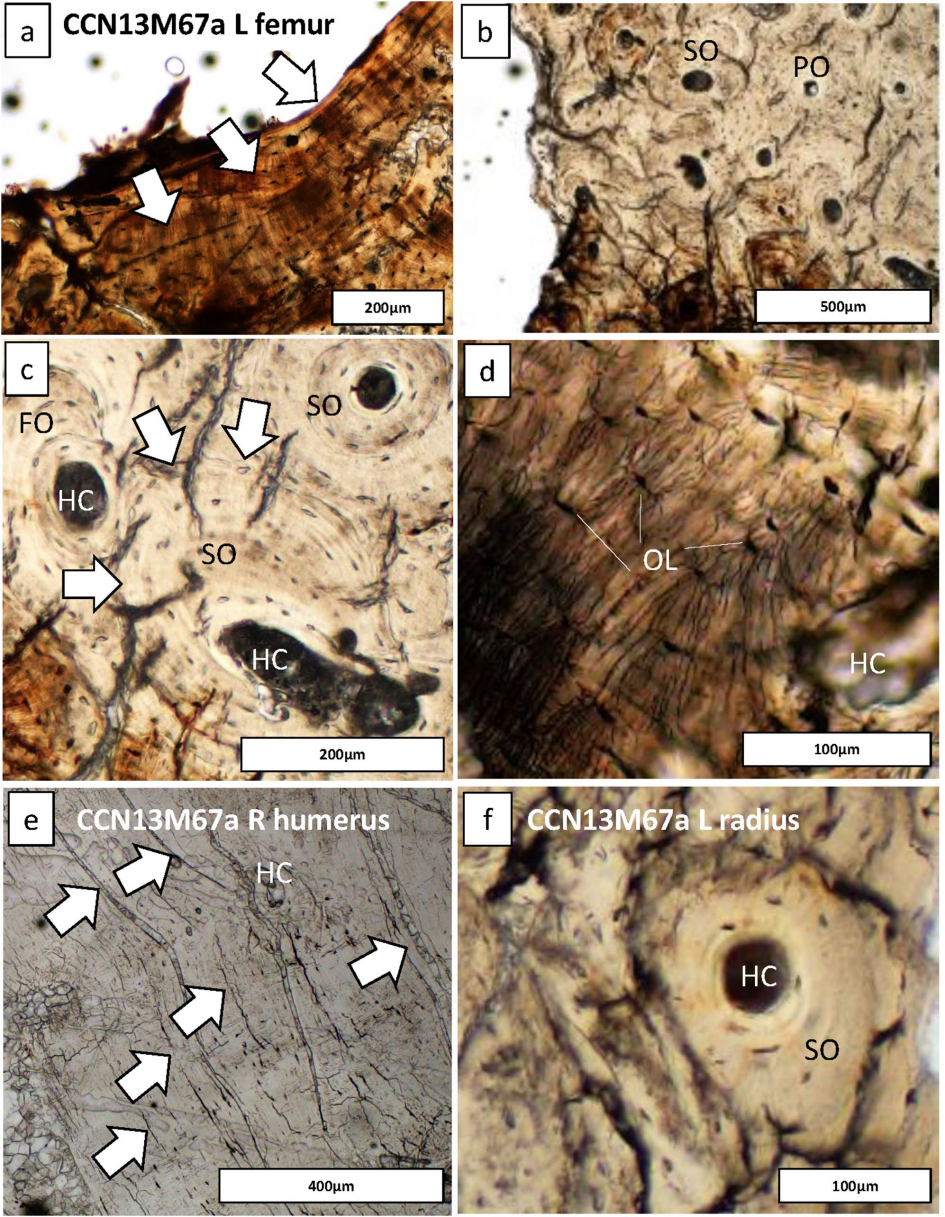
Regions of bone captured from the endocortical surface in the adolescent (CCN13M67a), allowing to examine the degree of bone modelling and remodeling. (**a**–**d**) The femur: Secondary osteons (SO), primary osteons (PO), Haversian canals (HC), and osteocyte lacunae (OL) can be seen. (**a**) White arrows point to endosteal lamellar layers which are typical for this bone region. (**c**) White arrows point to a cement line of a secondary osteon that indicates a remodeling event of a fragmentary osteon (FO) underneath, confirming cyclical replacement of old bone with new bone tissue, possibly driven by increased metabolic bone needs. (**e**) White arrows point to primary lamellar bone layers and an isolated HC. (**f**) A SO amongst primary bone.

In contrast, the bone samples in the young adult female (CCN13M40a), showed denser and enlarged endocortical surfaces. The adolescent (CCN13M67a) presented with a mix of large porous regions of the endocortex (right humerus) and regions of increased cortical density (femur). Possible beginnings of the ‘trabecularization effect’ such as those observed in the old adult male (CCN13M59a) were found in the right humerus of the old adult. The extent of medullary bone size reduction can be seen, particularly in the adolescent femur section where the cortical wall is unusually enlarged (Fig. 4d). This femur showed evidence for secondary bone remodeling confirming cyclical replacement of old bone with new bone tissue, possibly driven by increased metabolic bone needs (Fig. 6). There was a clear pattern whereby samples from the femur did not show evidence for widespread porosity and coalescing of bone pores, but were observed in the bones of the upper limb (ulna, humerus, and radius) (Fig. 6).

We compared the microscopic results to various diseases of infectious and metabolic etiologies known to cause the macroscopic ‘bone-within-a-bone’ sign (Supplementary text S1). The microscopic outcomes of Con Co Ngua are only consistent with clinical bone histological observations in cases of beta thalassemia. Increased osteoclast-mediated resorptive activity, and decreased osteoblastic activity have both been described as underlying processes that increase porosity of the endocortical margins of the long bones in beta thalassemia^21^. In thalassemia, skeletal changes can be localized, due to focal deposits of iron during iron overload which is consistent with the variation between the upper and lower limbs we observed across the samples^21–23^. The overall histological bone pattern observed in the Con Co Ngua individuals therefore supports localized metabolic changes consistent with thalassemia.

## Discussion

### What hemoglobinopathy variants were present in prehistoric Vietnam?

Our results demonstrate skeletal evidence for thalassemia from the early seventh millennium BP in northern Vietnam. The gross skeletal changes in all but one Man Bac individual are consistent with beta thalassemia^4^. The clinical consequences, including bone changes, of beta thalassemia only develop following replacement of fetal gamma hemoglobin with adult beta hemoglobin in the months following birth. However, the possible case diagnosed in a Man Bac newborn may be consistent with alpha thalassemia major (Bart’s hydrops fetalis)^24^. Alpha thalassemia major, caused by deletion of all of the four alpha alleles, is a common variant in Southeast Asia today^25^. There is little clinical description of the skeletal complications of alpha thalassemia major due to its fatal outcome. However, severe bone marrow expansions occur in utero during growth detected as early as 18 fetal weeks^7^, and skeletal complications similar to beta thalassemia major can be expected^24^. Severe growth stunting is a common outcome of alpha thalassemia major, and skeletal deformities have been clinically documented, but not described^5,7^.

Evidence for co-occurrence of both alpha and beta thalassemia variants at Man Bac is of evolutionary significance. The gross skeletal pathologies of Man Bac post-birth individuals are commonly observed in patients with beta thalassemia major, a severe form that requires removal of excess iron and blood transfusions in order to survive past infancy^26^. However, 57% (4/7) of individuals macroscopically diagnosed with thalassemia at Man Bac were older than 1-year of age, having survived infancy without treatment. Alpha and beta thalassemia co-inheritance is known to result in a milder form of disease than beta thalassemia major due to a balance of depleted alpha and beta hemoglobins^27^. Additionally, the Southeast Asian variant HbE beta thalassemia commonly results in severe forms of thalassemia that do not require blood transfusions to survive (known clinically as thalassemia intermedia)^28^. It is possible that co-inheritance of beta thalassemia with alpha and/or HbE may account for the severe skeletal changes in individuals at Man Bac who survived past infancy.

It is not possible to determine whether the Con Co Ngua individuals had beta or alpha thalassemia. While there is clear clinical recognition of the skeletal changes of beta thalassemia^29^, HbH alpha thalassemia caused by deletion of three of the four alpha alleles are reported to cause mild to moderate skeletal deformities in some patients^5^. Significant facial deformity was not recorded at Con Co Ngua. However, infants at this site were very poorly preserved, so more severe forms of thalassemia at Con Co Ngua cannot be ruled out.

### Evidence for deep antiquity of thalassemia and malaria in Mainland Southeast Asia

Based on our macroscopic and microscopic observations we suggest that thalassemia was potentially a considerable burden for Southeast Asian populations prior to the adoption of farming. This contrasts with our current knowledge on the emergence of thalassemias in the Mediterranean and demonstrates that the agricultural transition was not the defining factor in the emergence of this disease in Southeast Asia. We note here that the one adult with probable thalassemia from Man Bac had dental and skeletal affinity to Australo-Papuan populations, such as those from Con Co Ngua (and all foraging groups from Vietnam prior to agriculture). The inhabitants from the Neolithic central Thailand site of Khok Phanom Di where thalassemia was previously reported represent mixed Australo-Papuan and East Asian dental affinities^30^. Therefore, a deep antiquity for thalassemia in the region prior to the Neolithic appears highly probable. However, the complexity of gene flow and stabilizing selection in the region exceeds the capabilities of skeletal morphometric data.

The heterogeneity of mutations that cause beta, HbE beta and alpha thalassemia in Southeast Asia support multiple instances of independent emergence within the region likely tied to a consistent threat of malaria within MSEA^31,32^. Alpha thalassemia variants in Southeast Asia populations have similarly been identified in modern indigenous Papuan and Austroasiatic (modern Southeast Asian) speaking populations^33^. The widespread geography of these variants today, and the compounded effect of multiple migration and genetic admixture events since prehistory, mean it is not possible to determine the origins of these variants from the distribution in modern populations. However, different haplotypes do indicate separate founder effects in Australo-Papuan (indigenous Australian and New Guinean Highlanders) and Austroasiatic (Thai) groups^33^. At least in the case of alpha thalassemia, a pre-agricultural deep history of this disease in MSEA is consistent with present day genetic data. In contrast, selection models of one HbE variant from a Thai population indicates an emergence between 4400 and 1240 years ago, consistent with agricultural intensification between the Neolithic to Iron Age periods (4500–1500 BP) in MSEA^34^. HbE emergence is consistent with the time period of the Man Bac individuals with thalassemia. Multiple independent events of thalassemia emergence may have occurred due to different social transitions in Southeast Asia’s prehistory.

The presence of thalassemia in prehistoric MSEA, as is the case today, indicates a constant selection pressure of malaria stemming from deep antiquity. It is expected that the gene frequencies of thalassemias in the absence of malaria would not have been maintained given its mortal cost when inherited in its severe forms^14^. While *P. falciparum* has a considerably higher mortality rate than *P. vivax*, thereby incurring a stronger selection pressure, both *Plasmodium* species are endemic to the region and thought to have had possible selection effects on thalassemia in Southeast Asia^35^. Both *P. falciparum* and *P. vivax* malarias share African origins. It is thought that *P. vivax* emerged out of Africa with human groups prior to 30,000 years ago^36^ consistent with the first anatomically modern humans who inhabited MSEA as early as 60,000 years ago^16^. *P. falciparum* is thought to have emerged in Africa approximately 60,000–40,000 years ago, and estimated to have undergone a bottle neck approximately 6,000–4,000 years ago that likely favored human infection^37^. This proposed bottle neck event post-dates the skeletal evidence for thalassemia at Con Co Ngua and pre-dates that of Man Bac. Rather than provide resistance, alpha thalassemia has been proposed to increase susceptibility to infection by *P. vivax*^35^. This susceptibility may be an adaptive mechanism to cross vaccinate against *P. falciparum*^35^ which signals the complex connection between *Plasmodium* infections and the emergence of thalassemia. The association between thalassemias and *P. vivax* morbidity and mortality, however, remains unknown and is a necessary component to understanding the relationship between malarial types and the emergence of thalassemias in the region^38^. Rarer still, *Plasmodium* variants commonly found in Southeast Asian forest macaques have also been known to transfer to humans^39^, an important local reservoir in communities in Vietnam^40^. Potential human to non-human primate interactions may also factor into the relationship between malaria and thalassemia in the prehistory of the region. Both Man Bac and Con Co Ngua inhabitants exploited non-human primates as a food source^17,41^.

Lowland flood plains in Vietnam, such as where Man Bac and Con Co Ngua are located have low reported malarial cases and primary vectors today, as is the case with coastal and estuarine environments^42^. However, the original ecology of the region surrounding these sites in antiquity included movement within fringe forests, brackish water, and riverine areas that may have placed the inhabitants at risk of a number of different *Anopheles* mosquito species that carry both human and simian *Plasmodium* species^42^. In tropical forested areas of MSEA, malarial vectors are common. Tropical forested regions expanded substantially from approximately 14,000 years ago following the Last Glacial Maximum, which would have been opportunistic for the spread of species *A. dirus* and *A. minimus*, the primary mosquito vectors of malaria in MSEA^42^. The tropical climate would have enabled year-round transmission even in seasonal areas such as northern Vietnam that experience dry and wet seasons^43^. While pre-agricultural, the complex hunter-gathers of Con Co Ngua existed in large sedentary populations, thereby encompassing a suite of factors that have been argued to be associated with the emergence of malaria and thalassemia in the Mediterranean^44^. Sedentary residential mobility would have enabled completion of the cycle of the disease within one host population^42^, increasing the selection pressure on these populations. In Southeast Asia today, an increased risk is observed in people who live in villages but actively exploit the forest for resources^45^. This risk appears to be related to the early night feeding times of these vectors. The exposure to forest *Anopheles* mosquitos and sedentary residence alone would likely have been sufficient to increase the probability of contracting malaria in the absence of agricultural land clearing.

### The agricultural transition and gene flow variation

While Con Co Ngua individuals were less well preserved than those from Man Bac, 10% (7/70) of the latter assemblage presented evidence for thalassemia. Man Bac is contemporaneous with Khok Phanom Di indicating these alleles were widespread through the region at this point in time. Therefore, the transition to agriculture may have considerably altered the gene flow or increased the selection pressure of thalassemia mutations, and shaped the relationship between human groups and malarial vectors in the region. Wet rice agriculture (irrigation) was only developed during the Iron Age (2500–1500 BP) in Southeast Asia^46^. This form of agriculture has been previously, erroneously, argued to be linked to increased malarial vectors compared to dry rice agriculture in MSEA^47^. Our results indicate a strong relationship between human groups and malaria prior to wet rice farming. Present day reports of differences in malarial vector prevalence in irrigated versus non-irrigated farming localities vary and depends on other ecological dynamics such as seasonality^48^. In Mainland Southeast Asia, *A. dirus* is found in significantly higher densities in forested areas than in villages, and reside in shallow pools^42^ rather than in deep waters. Therefore, the moated settlements of the Iron Age in Thailand may have made the ecology around villages less attractive to primary malarial vectors or attracted different *Anopheles* mosquitos such as *A. barbirostris* with significantly lower vector potential^49^. Instead, the reliance of dry rice farming on mild seasonal flooding may have increased the exposure of Neolithic inhabitants to malarial vectors^42^, particularly as they continued to exploit forested areas through mixed farmer-foraging practices^50^. While Pre-Neolithic foragers in northern Vietnam and southern China were arguably primarily sedentary, an increase in sedentism and significant population growth during the transition to agriculture in MSEA^51,52^ would have increased the transmission potential within human populations.

Possible interaction between Man Bac inhabitants and other nearby contemporaneous sites further inland, where higher vector densities have been reported^42^, may have also contributed to the presence of thalassemia at Man Bac. Alternatively, we also recognize that gene flow of thalassemia alleles from other inland Neolithic sites may be responsible, and a direct relationship between thalassemia and malaria presence at Man Bac cannot be determined. A similar argument can be made for Con Co Ngua, as contemporaneous forager sites of northern Vietnam are also archaeologically documented to have maintained interactions through exchange^53^. Nevertheless, it is apparent that mobility and interactions within and between groups were essential for the spread of thalassemia variants in the prehistory of MSEA, and the prevalence of this disease can be tied directly or indirectly to malarial endemicity in prehistoric MSEA.

## Conclusion

Through a combined macroscopic and microscopic approach we demonstrate that thalassemia has been present in northern Vietnam from at least the early seventh millennium BP. This finding indicates that agriculture was not a crucial factor in the emergence of thalassemia in response to malaria in Southeast Asia as appears to be the case in the Mediterranean. In the context of large sedentary forager populations exploiting forested resources, such as the inhabitants of Con Co Ngua, we propose a pre-agricultural origin for the emergence of thalassemia in MSEA as an adaptive response to the threat of malaria. However, the agricultural transition approximately 4500–3500 years ago likely encouraged the spread of malarial vectors, increasing the gene frequencies of thalassemias. The outcomes of our research indicate a deep history of thalassemias which are endemic in MSEA today.

## Methods and materials

### Ethics and approvals

Paleopathological observation and collection and analysis of samples for histological investigation was approved by the Vietnam Institute of Archaeology in Hanoi, Vietnam on October 12, 2018. Bone samples were exported to the Australian National University in Canberra under approval by Dr. Nguyen Gia Doi (Director), and are currently housed at this institution until further notice. As the samples are of an archaeological nature, no ethical approvals were required for this study.

### The sample

We macroscopically assessed 70 individuals from Man Bac and 155 individuals from Con Co Ngua for pathology. All skeletal elements with pathological change with the potential for diagnosis were radiographed. Three individuals from Con Co Ngua were sampled for histological analysis.

The skeletal assemblage of Man Bac was well preserved. While post-mortem breaks were frequent, the skeletons were easily reconstructed. Surfaces were excellently preserved. For most skeletons both adult and subadult were either complete or near complete^54^. In contrast, the individuals from Con Co Ngua presented with erosion of the cortical surfaces, but not to the degree that pathologies could not be observed. Post-mortem breakage was common, and a large proportion of the assemblage was at most partially complete. Subadults were commonly poorly preserved, with infants highly fragmented. Methods for age and sex of the individuals from Man Bac and Con Co Ngua were previously reported and briefly summarized here^50,55,56^. Non-metric traits of the os coxae, cranial morphology, and sample-specific post-cranial functions were used to estimate adult sex^57–60^. Adult age-at-death was estimated by way of pubic symphyseal morphology^61^ and/or sample-specific molar wear functions developed by Oxenham^60^. Subadult age-at-death was estimated using dental eruption and calcification standards^62,63^, and/or skeletal maturity schedules^64^.

### Paleopathological differential diagnosis

The paleopathological diagnosis of thalassemia was based on macroscopic and radiographic observations of pathologies in dry bone. A standardized ‘threshold’ diagnostic protocol for thalassemia was produced following clinical diagnostic standards (Table 2). A possible case was diagnosed when an individual exhibited one diagnostic or two suggestive lesions. A probable case was diagnosed when an individual exhibited one strongly diagnostic or two diagnostic lesions. Such recording methods are consistent with a number of previously published diagnostic protocols in paleopathology, and ensure diagnostic rigour^18,65^.

**Table 2:** Criteria for diagnosis of thalassemia in dry bone (SD = strongly diagnostic, D = diagnostic, and S = suggestive).

Pathology	Diagnostic strength	Differential diagnosis	References
Marrow hyperplasia of the facial bones: maxillae- leading to ventral displacement of central incisors, zygomatic bones-leading to orbital displacement, and/ or mandible (rodent facies deformity)	SD		^29,66–70^
Radiographic: “rib-within-a-rib” appearance. Radiographically defined sclerotic bands within the ribs due to extramedullary hematopoiesis	SD	Sickle cell anemia, osteomyelitis, leukemia*	^29,66,68,71^
Poor or lack of pneumatization of the paranasal and cranial sinuses sparing the ethmoid sinuses	D	Neoplasms, Paget’s disease, trauma, hypopituitarism, hypothyroidism, osteopetrosis, sickle cell anemia	^29,68^
Widening of entire rib, or widening of the rib head and neck with pronounced bulbous appearance posteriorly (costal osteomas). Associated with radiograph appearance of erosion of the inner cortex	D	Neuroblastoma, Nieman-Pick disease, Leukemia	^29,66,68,71^
Enlarged tubular bones of the hands and feet due to marrow hyperplasia (infants) sometimes associated with enlarged nutrient foramina *or* Radiographic: coarse trabecular patterns of the hands or feet, sometimes associated with cyst-like lucencies due to focal collection of hyperplasic marrow	D	Treponemal disease, leprosy, tuberculosis	^12,29,66,68^
Premature fusion of epiphyseal plates particularly of the proximal humerus and distal femur, often causing short long bone maximum length	D	Scurvy, hypervitaminosis A, trauma, achondroplasia, Morquio’s disease, Ellis-van Creveld disease, peripheral dysostosis, poliomyelitis, mucopolysaccharidosis, rickets, osteomalacia	^12,29,66,68,72^
Extensive marrow proliferation of the long bones leading to expansion of the medullary canal, associated with thin cortices (and in extreme circumstances honeycomb-like porosity) resulting in swollen appearance or metaphyseal flasked shaped deformities	S	Other hemolytic anemia, scurvy, rickets, metaphyseal dysplasia, Gaucher’s disease, osteomyelitis	^66,68,73^
Severe porotic hyperostosis/ diploic expansions of vault and maxilla with “hair-on-end” appearance and/ or cribra orbitalia	S	After Lagia^74^: hemolytic anemias and red cell enzyme disorders (including sickle cell disease, iron deficiency anemia and G-6-PD deficiency), cancers (including leukemia, multiple myeloma, meningioma, metastases and secondary to kidney cancers), and polycythemia	^29,66,68,74^
Wide dental spacing	S	Skeletal dysplasias, normal variation	^66^
Spiculated or scalloped proliferation of subperiosteal reactive new bone on the shafts of the limb bones and the clavicles	S	Infectious diseases, rickets, scurvy, hypertrophic osteoarthropathy, Gaucher’s diseases, Paget’s disease	^12,69,75^
Marked osteoporosis and cortical thinning of the vertebrae, with compression fractures in severe cases	S	Age related osteoporosis, osteomalacia, scurvy, trauma	^29^
Bone infarction	S	Osteomyelitis, sickle cell anemia, osteosarcoma	^76^
Enlargement and alteration of the trabecular pattern in flat bones (pelvis and scapula)	S	Other hemolytic anemia, leukemia	^70^

A *probable* case is defined as an individual exhibiting at minimum one strongly diagnostic pathology *or* two diagnostic pathologies. A *possible* case is defined as an individual exhibiting at minimum one diagnostic *or* two suggestive pathologies.

*A strongly diagnostic lesion is one that is considered pathognomonic for that disease and alone stands as evidence of probable disease. In extremely rare instances these pathologies can occur in other diseases which are listed here in the differential diagnosis.

Gross skeletal manifestations of thalassemia are restricted to beta thalassemia only, except in the case of alpha thalassemia major (also called *Bart’s hydrops fetalis*) a fatal form, where infants die prior to or immediately following birth^7^. Severe porotic hyperostosis leading to a ‘hair-on-end’ appearance is a common skeletal manifestation of thalassemia (as is the case in other anemias) and has been reported particularly in cases of thalassemia major^68,77^.

Extensive marrow hyperplasia of the medullary canal and within cancellous bone regions cause considerable thinning of the bone cortex, and expansion of the area of the medullary canal^68^. The extent of the marrow hyperplasia causes thinning trabeculae. However, subperiosteal new bone response to the trabecular destruction instigates the coarsening of trabeculae which is observed radiographically^68^. In extreme circumstances cortical thinning can progress to destruction of the cortex and subsequent honeycomb appearance of the bone surface^70^.

A distinct facial deformity called rodent facies occurs in severe cases particularly in thalassemia major whereby extensive marrow proliferation of the maxillae and zygomatic bones creates anterior bulging of the face, often associated with anterior displacement of the central incisors, and lack of pneumatization of the maxillary, sphenoidal and frontal sinuses^29,66–68^. This pathological lesion is strongly diagnostic for thalassemia, particularly of thalassemia major (Table 2)^29,68^. The marrow proliferation also causes anterior teeth protrusion and malocclusion of the remaining teeth^66^. A strongly diagnostic radiographic feature of this disorder is the ‘rib-within-in-a-rib’ sign. This feature is produced by extensive marrow hypertrophy perforating the thin cortex of the rib associated with a radiodense line in the middle of the medullary canal^29,66,69,78^. Hypertrophic changes to the hands and feet of infants (with  associated expanded foramina in the phalanges) is also characteristic of the disease, as this lesion indicates an extreme form of anemia unlikely to be associated with non-genetic etiologies^12,68^.

Marrow perforation of the cortex known as extramedullary hematopoiesis occurs. In certain circumstances subperiosteal new bone response similar to the “hair-on-end” appearance of the skull, surrounding thin cortices of postcranial bones, can result as a consequence of marrow perforation^69,75^. This skeletal response has been documented to lead to premature fusion of the epiphyses of long bones particularly of the proximal humerus and distal femur^68,75^. The extent of prematurity is dependent on the individual. This lesion is more common in cases of thalassemia intermedia^75^. Bone infarction, while less common in thalassemia than sickle cell anemia, has also been reported. In this pathological condition, bone death occurs followed by inflammatory new bone response around the region of necrosis, and has a distinct appearance on radiographs^76^.

### Histological sectioning

All sectioning procedures followed standard protocols^79,80^ and were completed at the Institute of Archaeology, Hanoi, Vietnam. A Dremel Variable-Speed Rotary Tool 3000 with associated Dremel blades was used to cut into the bone shafts. This involved making two longitudinally oriented parallel (along the long bone axis) cuts, followed by a transversely oriented (perpendicular to the longitudinal cuts) cut, so that sections freely detached themselves from the bone. Permissions obtained from the collection curators strictly stipulated that sections were only to be removed from the bone regions already affected by post-mortem breaks to avoid further damage to the specimens. For example, where the long bone was broken in half or had epiphyseal ends missing due to taphonomic issues. Sampling in those regions avoided further damage to the specimens. However, we ensured that the bone sections still derived from the long bone shafts so that we could assess the histological changes on the endocortical surfaces. The three individuals sampled for histological analysis met these criteria. We sampled the left and right femur of CCN13M40a (Fig. 4). The right humerus, left ulna and left radius of CCN13M59a were sampled as they were the only available long bones for this individual (the lower limb only consisted of a fragmented right fibula). The right humerus, left radius, and left femur of the subadult CCN13M67a were sectioned as both the upper and lower limb bones showed a degree of endocortical thickening. The lower (distal direction) third of the left femoral shaft, approximately 4 cm below the linea aspera, and the midshaft of the right femur, were sampled in CCN13M40a. The upper limb bones of CCN13M59a were sectioned at the distal end of the left ulna and the right radius, and the midshaft of the right humerus. The left femur of CCN13M67a was sectioned at the middle of the upper (proximal direction) third of shaft, the distal third of the shaft in the left radius, and the upper (proximal direction) third shaft of the right humerus. We note that the left radius in this individual shows a well remodeled and healed fracture at midshaft (Supplementary Fig. S2), which clearly resulted in a deformation of the bone. This will need to be borne in mind when interpreting the appearance of histology in thin section. A total of eight, approximately 1 cm thick, roughly C-shaped, sections of bone from the posterior aspect were removed for the entire sample (Fig. 4).

Prior to histological preparation, external size measurements of the bones and sections were recorded using standard digital calipers and a soft measuring tape. All measurements were repeated at least two times from which an average was computed and reported here. The measurements^79^ were: shaft circumference (Circ) at point of sectioning (mm), anterior–posterior diameter in mm (AP dm), medial–lateral diameter in mm (ML dm), cortical width (Ct.W) of the extracted sample measured from both the proximal and distal surfaces in the lateral direction (from the endosteal, referring to the endosteum lining the medullary cavity, to the periosteal surfaces).

### Histopathology

No prior information on age, sex or macropathology was provided for the samples to ensure an objective observation. All samples were processed into thin sections following standard methods applicable for histological analysis of archaeological human bone^81,82^. The processing was completed at the Hard Tissue Histology laboratory in the School of Archaeology and Anthropology at the Australian National University (Canberra, Australia), which is also where the resulting thin sections are curated until further notice. Each sample was embedded in Buehler epoxy resin, cut on a low-speed saw equipped with a diamond wafering blade, and attached to microscope glass slides using Stuck epoxy glue. The attached samples were further trimmed on a cutting saw, and later lapped on a series of grinding pads of various grit sizes that increased from 400 to 1200 in coarseness. The thickness of each section was controlled using a handheld Buehler 28 × 48 mm target holder, allowing to produce sections of approximately 90–100 μm thickness. The sections were polished using a Buehler MicroPolish powder, 0.05 µm, until all scratches created during grinding were removed. This was followed by cleaning the sections in an ultrasonic bath, dehydrating them in a series of ethanol baths, and clearing using xylene for histopathology. The samples were finally covered with glass cover slips glued to the bone surface with DPX.

### Imaging and histomorphological analysis

Each thin section was imaged using transmitted and polarized light under a high powered Olympus BX53 microscope equipped with a DP74 high resolution camera^81^. We used a series of objective lenses, ranging from 4 × to 40 × (40 × to 400 × total magnification), to examine histological features^82^. Each section was first examined for any overall diagenetic damage, and second for patterns in, and areas of, abnormal bone matrices and micro-organization. This was followed by a targeted examination of bone regions on the endocortical surfaces, which is where we hypothesized abnormal changes would occur as per the macroscopic manifestation (Fig. 4). As the border between intra-cortical and endocortical bone relies on a somewhat arbitrary division, the endocortical area examined in our samples was, on average, 1.5–1.7 cm once the entire section surface was sub-divided into four equal segments (one sub-periosteal, two intra-cortically, and one endocortically). We captured individual regions of interest (ROIs) from within the endocortical areas. It is also where we recorded a full endocortical strip of bone using an automated stitching tool of the Olympus CellSens software.

The microscopic examination revealed an overall moderately good preservation of the samples^83^. Our histological examination was based on inspecting bone regions for: bone matrix type (woven, lamellar)^84^; bone growth stage (primary, secondary/ Haversian)^85^; bone remodeling indicators (poorly remodeled with singular secondary osteon units, well remodeled with several generations of fragmentary and intact secondary osteons)^80,85^, ‘giant’ resorption canals (porosity effect indicating prolonged osteoclast-mediated resorption, tabularization effect)^86,87^. Where necessary, we also undertook histomorphometric measurements using ImageJ ‘straight line’ tool to obtain maximum diameter of pores in cases of abnormal porosity.

## Supplementary Information

Supplementary Information 1.

Supplementary Information 2.

Supplementary Information 3.

Supplementary Information 4.
