# Supplementary figures and images for: Forager and farmer evolutionary adaptations to malaria evidenced by 7000 years of thalassemia in Southeast Asia
Source: Sci Rep. 2021 Mar 11;11:5677. doi: 10.1038/s41598-021-83978-4 (PMC7952380; doi:10.1038/s41598-021-83978-4)

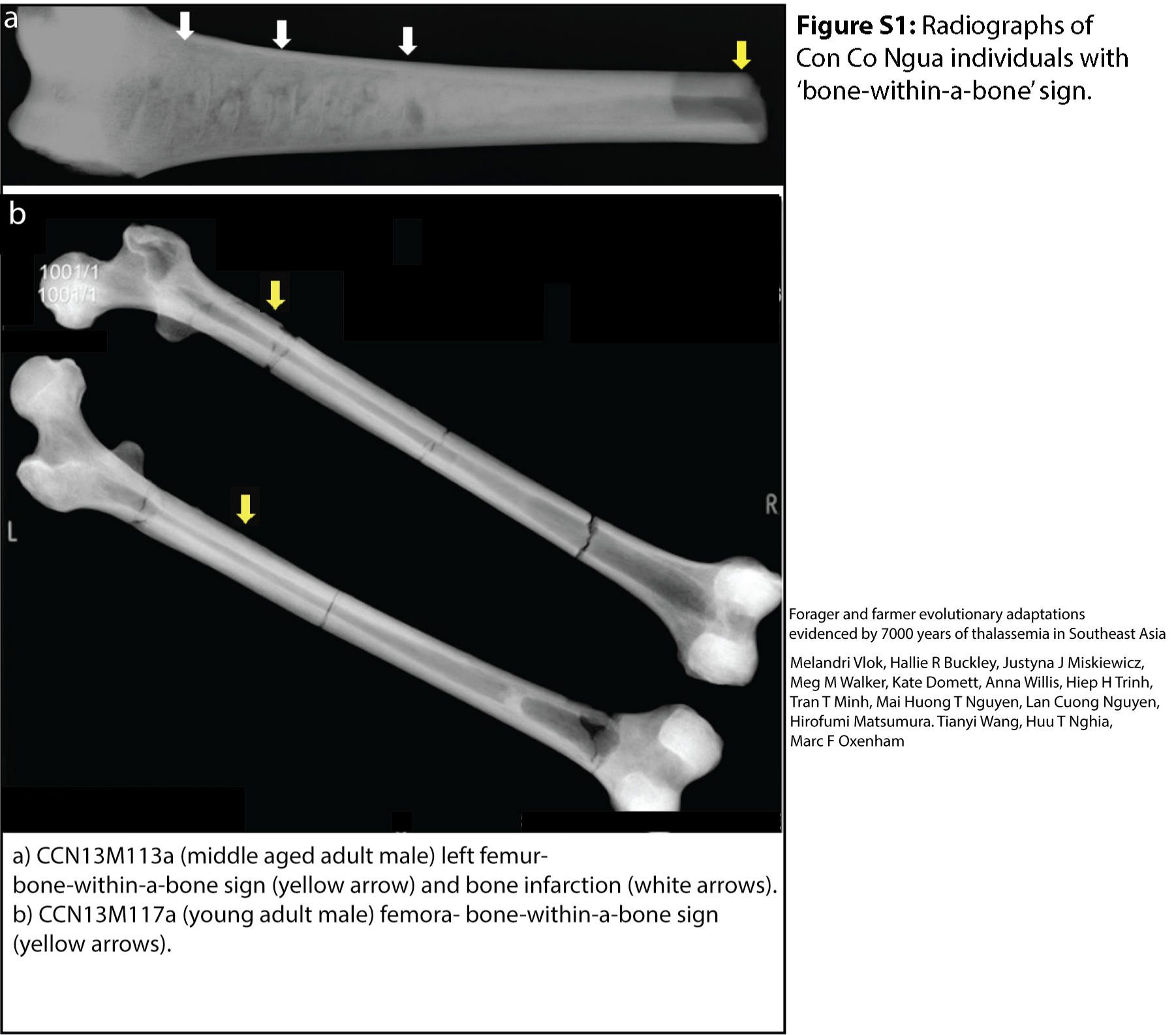

Supplement: Supplementary file 1 — Supplementary Information 1. [file 41598_2021_83978_MOESM1_ESM.tif]

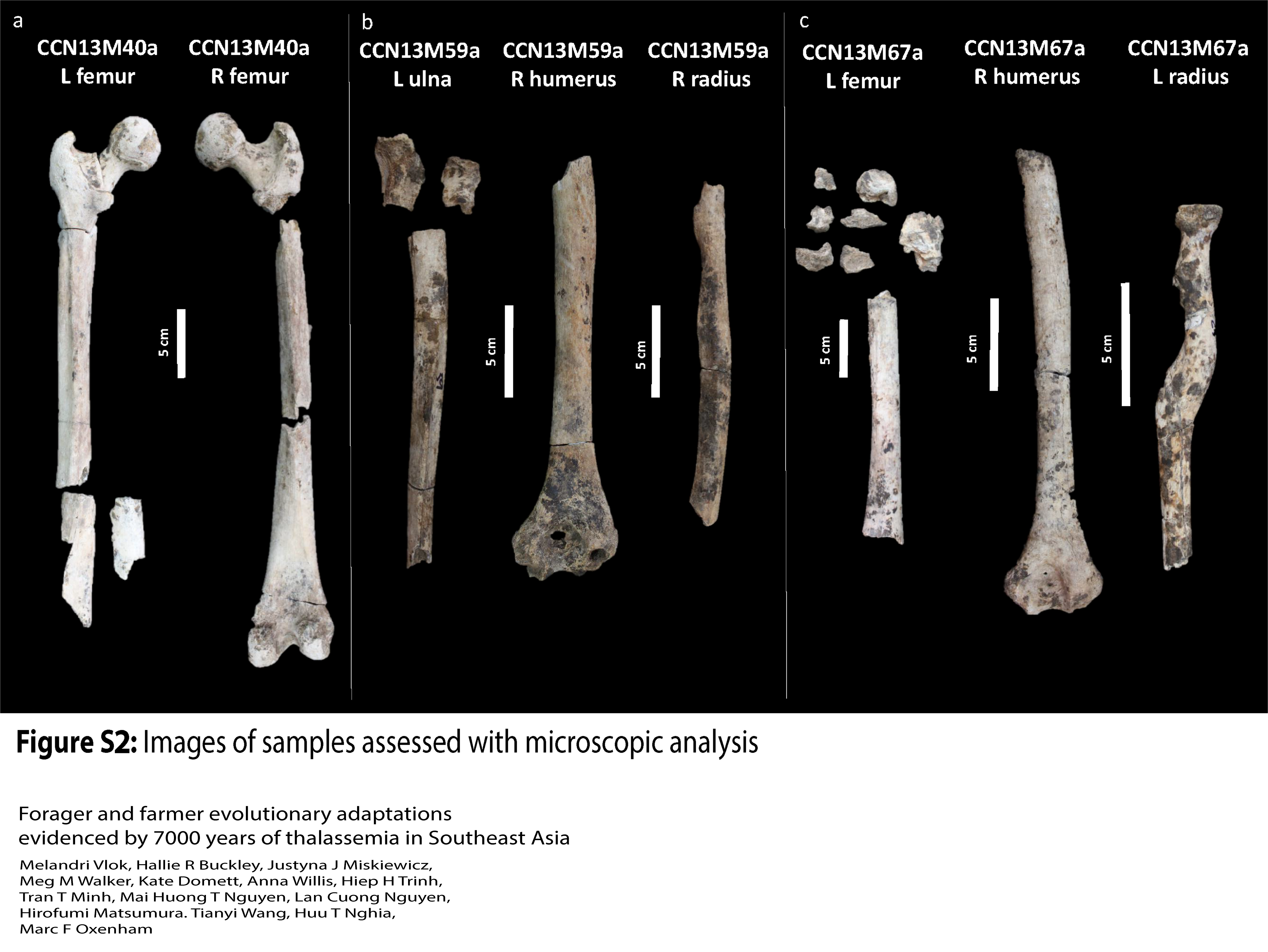

Supplement: Supplementary file 2 — Supplementary Information 2. [file 41598_2021_83978_MOESM2_ESM.tif]
