# Supplementary material for: Forager and farmer evolutionary adaptations to malaria evidenced by 7000 years of thalassemia in Southeast Asia
Source: Sci Rep. 2021 Mar 11;11:5677. doi: 10.1038/s41598-021-83978-4 (PMC7952380; doi:10.1038/s41598-021-83978-4)
Supplement: Supplementary file 3 — Supplementary Information 3. [file 41598_2021_83978_MOESM3_ESM.docx]

**Forager and farmer evolutionary adaptations to malaria evidenced by 7000 years of thalassemia in Southeast Asia**

Melandri Vlok, Hallie R Buckley_,_ Justyna J Miszkiewicz, Meg M Walker, Kate Domett, Anna Willis, Hiep H Trinh, Tran T Minh, Nguyen T Mai Huong, Nguyen Lan Cuong, Hirofumi Matsumura, Wang Tianyi, Nghia T Huu , Marc F Oxenham

**Supplementary Text S1: Differential diagnosis and outcomes of macroscopic and microscopic analyses**

**Man Bac differential diagnosis**

Applying the diagnostic protocol for diagnosis of thalassaemia in dry bone we identified one individual that exhibited skeletal pathology consistent with possible, and six individuals consistent with a probable case of thalassaemia (10%, 7/70). Four subadults aged approximately 6 months to 12 years exhibited marrow hyperplasia of the zygomatic bones, maxilla and/or mandible consistent with *rodent facies*, a skeletal change pathognomonic for beta thalassaemia (Fig. 2). Rodent facies was associated with crowding and anterior protrusion of the anterior maxillary teeth in this individual. Radiographs confirm marrow hyperplasia of the facial bones, strongly diagnostic for thalassaemia. Additionally, lack of pneumatisation (development) of the paranasal and frontal sinuses were present in two individuals (Fig. 2). Three individuals also presented with radiographic “rib-within-a-rib” sign (Fig. 3) again strongly diagnostic for thalassaemia, and three individuals exhibited severe porosity of the orbits known as *cribra orbitalia* (Fig. 2). Diploic expansion was not associated with porosity of the ectocrania. However, in two individuals, “hair-on-end” endocranial lesions were also present, similar to changes commonly documented on the ectocrania of thalassaemia patients (Fig. 2). Limb bones exhibited marrow hyperplasia associated with cortical thinning (rarefaction) or spiculated diffuse subperiosteal new bone (extramedullary haematopoiesis). Only one adult was identified with pathology consistent with thalassemia (ID: MB07H1M8, middle aged adult male). This individual exhibited radiographic “rib-within-a-rib” sign as well as enlarged foramina of the phalanges. Marrow hyperplasia of the phalanges was observed on the radiographs (Fig. 3). A neonate with diploic expansion of the temporal bone also exhibited enlarged scapulae and ilia. These elements presented with alteration in the structure of the trabeculae consistent with thalassaemia. This neonate constitutes the only possible case identified in the assemblage.

**Con Co Ngua differential diagnosis**

Seven individuals (4.5%, 7/155) exhibited macroscopic and/or radiographic “bone-within-a-bone” signs that did not significantly alter the contours of the external cortical margins (Supplementary Fig. S1; Supplementary Table S1). These pathologies are synonymous with macroscopic subperiosteal new bone on the shafts of limb bones due to extramedullary haematopoiesis in thalassaemia, as was observed in the Man Bac individuals diagnosed with the disease, but can also be caused by chronic infection (osteomyelitis, tuberculosis and treponematosis), scurvy, Gaucher’s disease, Paget’s disease, osteopetrosis, hypertrophic osteoarthropathy or residual rickets^1^. This lesion is therefore a suggestive trait, and alone is not diagnostic for thalassaemia. However, these individuals were also observed to have either medullary stenosis (partial closure of the medullary canal due to enlargement of bone on the endo-cortical surface), or widening of the medullary canal in multiple limb elements (Fig. 4d). No diagnostic signs of rickets such as cupping, flaring and fraying of metaphyseal plates of subadults was observed in CCN, and is not known to cause medullary stenosis. Scurvy is not likely to result in bone-within-a-bone radiographic signs in adolescents and adults^1^. Bone infection could be assumed to cause extensive macroscopic changes to the external cortices of the long bones^2^, particularly as multiple skeletal elements were observed to have “bone-within-a-bone” pathologies suggesting an advanced stage of pathology. Similarly, macroscopic changes to the external cortices of the bones are common findings in the remaining pathologies^3,4^. This was not the case in the Con Co Ngua individuals. While widening of the medullary canal is commonly associated with thalassaemia, medullary stenosis can occur secondary to ischemic necrosis (bone infarction)^5^. CCN13M113a, a middle aged adult male, also exhibited radiographic evidence for ischemic necrosis of the trabeculae of the distal femur, another suggestive trait indicating the presence of possible thalassaemia at Con Co Ngua in at least one individual, following the palaeopathological diagnostic protocol for thalassaemia (Table 3). The fragmentary nature of Con Co Ngua individuals made the identification of thalassaemia difficult. The disease is well known to contribute to poor preservation^6^. Thus, palaeohistological samples of three individuals from Con Co Ngua exhibiting bone within a bone sign and medullary stenosis were extracted (CCN13M59a, CCN13M40a and CCN13M67a) for further analysis.

**Palaeohistology Results**

Table 1 provides a summary of all external bone size measurements. Table 2 summarises the histological findings in all individuals and bones, focusing on the endo-cortical strip only.

**Table 1**. External bone morphometric data at the sampling location (Circ, APdm, MLdm) and of the sample extracted for histology (Ct.W). L: left bone, R: right bone, Circ: circumference of shaft at sampling location in mm, AP dm: anterior-posterior shaft diameter in mm, ML dm: medial-lateral shaft diameter in mm, Ct.W: cortical width in mm, N/A: the sample was too fragmented to obtain a meaningful measurement. *this measurement included deteriorated bone surface, so may not reflect true bone thickness

| **Con Co Ngua ID** | **Bone** | **Circ** | **AP dm** | **ML dm** | **Ct.W** |
| --- | --- | --- | --- | --- | --- |
| CCN13M40a | L femur | 77 | 26.51 | 23.74 | 8.245 |
|  | R femur | 78 | 26.24 | 22.79 | 8.04 |
| CCN13M59a | R humerus | 71.67 | 24.14 | 21.94 | 4.54 |
|  | L ulna | 42 | 12.68 | 14.43 | 3.74 |
|  | R radius | 45.33 | 12.40 | 16.16 | 3.58 |
| CCN13M67a (subadult) | R humerus | 65.5 | 19.02 | 22.16 | 5.53* |
|  | L radius | 44 | 12.22 | 13.80 | N/A |
|  | L femur | 77.5 | 25.93 | 24.14 | 9.06 |

**Table 2**. Summary of histological observations on the endo-cortical strip of bone. *indicates bones with substantial diagenesis of microstructure and thus cautious descriptions.

| **ID** | **Bone** | **Collagen matrix (lamellar, woven bone)** | **Predominant bone type (primary, secondary)** | **Remodelling (early phase/ moderate/ advanced)** | **Porosity/ prolonged resorption** | **Trabecularisation** |
| --- | --- | --- | --- | --- | --- | --- |
| CCN13M40a | L femur | Lamellar | Secondary | Possibly* several generations of secondary osteons (advanced) | Localised and restricted to lateral 1/3 of endo-cortex | No |
|  | R femur | Lamellar | Secondary | Possibly* several generations of secondary osteons (advanced) | Localised and restricted to middle 1/3 of endo-cortex | No |
| CCN13M59a | R humerus | Lamellar | Secondary | Possibly* secondary osteons only (moderately remodelled) | Severe and widespread well into the intra-cortical bone, giant coalescing pores evident | Yes |
|  | L ulna | Lamellar | Secondary | Possibly* secondary osteons only (moderately remodelled) | Severe and widespread, giant coalescing pores evident | Yes |
|  | R radius | Lamellar | Secondary | Possibly* secondary osteons only (moderately remodelled) | Severe and widespread well into intra-cortex, giant coalescing pores evident | Yes |
| CCN13M67a (subadult) | R humerus | Lamellar | Primary and secondary | Isolated secondary osteons, multiple primary vessels (early phases of remodelling) | Localised and restricted to middle and lateral 1/3 of endo-cortex | Possibly beginning |
|  | L radius | Lamellar | Primary and secondary | Isolated secondary osteons, some primary vessels (early phases of remodelling) | None | No |
|  | L femur | Lamellar | Primary and secondary | Multiple secondary osteons, fewer primary vessels (moderately remodelled) | Restricted, single trabeculae-like structure made up of endosteal lamellae, which appears normal | No |

CCN13M40a- Young Adult Female

The two femoral samples in this individual appear somewhat similar in their microscopic expression whereby both show compact secondary bone with localised and restricted porosity-like regions of the endo-cortical strip. However, these regions are inconsistently restricted to the lateral third in the left femur, and to the middle third in the right femur. There is no distinct trabecularisation effect in neither sample. The porous-like region in the left femur appears to show rod-like bone which resembles trabeculae off the medullary cavity and endosteal lamellae, which do not appear pathological. The cortical wall width (measured from the endosteal to the periosteal points) is slightly greater than that of the left sample, indicating some asymmetry in the two bones. The porosity restricted to the mid-portion of the endo-cortex in the right femur, on the other hand, shows enlarged and irregularly shaped canals encased within compact bone matrix which could indicate prolonged osteoclast-mediated resorption. While the left sample has a slight thicker cortical wall (Table 1), this is due to two different locations at shaft.

CCN13M59a- Old Adult Male

All three samples in this individual show extremely advanced porosity and indicators of prolonged osteoclast-mediated bone resorption on the endo-cortical surfaces. The porosity is widespread and consists of “giant” pores that appear to have been created as a result of adjacent coalescing pores (Fig. 4). In both the humerus and the ulna, the abnormal pores are irregularly shaped and somewhat flattened whereby they run along the transverse axis of the bone from the medial to lateral ends of the section (Fig. 4**)**. The maximum diameter of the largest pore in the humerus is 2314.72 μm, whereas it is 1515.29 μm in the ulna, which is extremely abnormal considering healthy Haversian canals that measure between 50-70 μm and only reach > 360 μm in non-trabecularised but pathological cases^7^. However, the extent of irregularity of the pore shape in the radius is not as advanced. There, the pores are rounder and less elongated, and measure maximum 1273.837 μm (Fig. 4). The extreme porosity in all three cases has resulted in the trabecularisation effect whereby cortical bone transforms into trabecular like structure separated by rodlike and irregular bone segments. This effect can be estimated to have impacted more than just the endo-cortical bone, whereby it extends for approximately 1.74 mm, 1.91 mm, and 2.92 mm in the ulna, radius, and humerus respectively.

CCN13M67a- Adolescent of unknown sex

This individual showed the most inconsistency in the microstructural expression of the endo-cortical bone whereby some samples showed evidence for prolonged osteoclast-mediated resorption, but others showed the opposite – compact and typical secondary Haversian bone matrix (Fig. 6). Given that this is a subadult, this individual also showed a combination of primary and secondary bone^8^. Only the right humerus in this individual shows endo-cortical porosity that appears abnormal. However, it is nowhere near as advanced as it is in CCN13M59a, with only an approximate 0.89 mm of endo-cortical bone affected and no evidence of porosity spreading well into the intra-cortical region of bone. This possibly indicates the beginning of a *trabecularisation* effect. The maximum diameter of pores is 1217.71 μm, which is also less than those recorded in the bones of CCN13M59a. On the contrary, the endo-cortical surface of the radius shows no abnormal remodelling or porosity at all. There is widespread primary lamellar bone apparent punctuated with isolated secondary osteons and multiple primary canals, consistent with a subadult developmental stage where we would expect a combination of both^8,9^. Finally, the femur shows well compacted and moderately remodelled bone (relative to the early phases of remodelling in the radius), with evidence of at least one earlier generation of concentric lamellae remodelled by subsequent events (Fig. 6). Primary vessels are still seen amongst the secondary bone as well. The only possible indication of non-compact bone on the endo-cortical surface is an isolated and restricted, single trabeculae-like structure that appears to be made up of endosteal lamellae. This is not abnormal, but typical of medullary cavity sporadic rod-like bony protrusions.

Combined macro and microscopic differential diagnosis Con Co Ngua

We explore different diseases which can cause macroscopic pathological changes observed at Con Co Ngua and discuss their clinical histopathological observations (osteomyelitis, tuberculosis and treponematosis, Gauchers’s disease, Paget’s disease, osteopetrosis, hypertrophic osteoarthropathy, residual rickets and thalassaemia). Lamm et al.^10^ recorded histopathology of osteomyelitis and observed trabecularisation and cortical porosity of the femoral shafts of 22 individuals aged 56 to 102 years, associated with cortical thinning. However, the cortical porosity was observed throughout the shaft and embedded within a clear involucrum as a result of an inflammatory process with infection. This is not the case in the samples analysed here where trabecularisation and increased porosity is restricted to the endosteal surface. Additionally, there is evidence of prolonged osteoclast resorption in the Con Co Ngua samples, which suggests osteoporosis-like deterioration of bone micro-architecture. Lamm et al. did not observe evidence for remodelling of the endo-cortical surfaces in healed cases of osteomyelitis. Schultz and Schmidt-Schultz ^11^ analysed specimens inflicted by tuberculosis in archaeological dry bone and fresh modern human bone tissue. While appositional lamellar growth was observed, bone remodelling did not appear to be directly impacted in the tuberculosis cases, and there were no obvious irregularities in the intra-cortical remodelling activity along long bone shafts. A lack of resorption along the endo-cortical margin does not align with the histology observed in our study. Resorption of the cortical, endo-cortical and trabecular matrix is not a product of treponematosis either^12^. An infectious origin to the pathological changes in Con Co Ngua can be ruled out.

Rickets and osteomalacia are bone mineralisation disorders that lead to the cessation of, or reduction in, osteoid mineralisation during modelling and remodelling^13^. Active rickets, a childhood disease, causes general osteopenia, and thinning of the cortices and trabecular bone^4^. In healed instances (residual rickets in adults), the accumulated osteoid is mineralised and the coarse trabecular structures are infilled, resulting in cortical thickening and clearly defined cortex. Given the advanced remodelled external cortices of Con Co Ngua individuals, the severe endocortical porosity is not consistent with a healed stage rickets. A similar process as active rickets is observed in adults with primary osteomalacia. However, again no micropathological changes associated with this metabolic condition was observed in any Con Co Ngua individuals. Primary osteomalacia does not result in localised histological changes as observed in the Con Co Ngua individuals with variable bone remodelling patterns in different samples across their skeletons.

Osteopetrosis is a result of a group of disorders that impede osteoclastic activity^14,15^. A s such, there are no similarities between the hard tissue histology of individuals with osteopetrosis and the individuals presented in this study ^14,15^. Diagnosis of hypertrophic osteoarthropathy (HOA) from dry bone histological techniques is difficult as changes are similar to those caused by haemorrhagic processes^12^. While increased trabecularisation has been documented, there has been no association with intracortical or endosteal bone resorption in HOA^2,16,17^.

Paget’s disease is a condition caused by disordered bone metabolism. Three stages of bone cellular activity occur in the progression of Paget’s disease of the bone: 1) osteolytic, 2) active and 3) blastic phases ^18,19^. Abnormal cell size and proliferation produces increased resorption through the cortex and results in thin trabeculae in the lytic stage. Increased size and activity of osteoclasts and osteoblasts during the blastic stage results in enlarged osteocyte lacunae and a weakened and disorganised collagen and tissue deposition mimicking a woven bone structure ^18,19^. This ‘mosaic’ like structure is not observed across the samples presented here but has been observed in other archaeological individuals^20^.

Although the lytic stage of Paget’s disease somewhat reflects the histology of individuals from Con Co Ngua, stage 1 is not pathognomonic and the thinned trabeculae associated with this stage are not present in the tissue samples presented here.

Gaucher’s disease can cause widespread or localised osteopenia of the cortices and trabeculae due to abnormal marrow cells and subsequent marrow expansion^21^. Additionally, osteosclerosis as a consequence of bone infarction can also occur in this disease^21^. Therefore, the disease somewhat fits the histological profile of Con Co Ngua individuals. However, the Con Co Ngua individuals do not exhibit the skeletal deformities observed in patients with Gaucher’s disease. Skeletal changes more commonly occur in the metaphyseal and epiphyseal regions rather than the diaphysis of long bones^22^. This disease is extremely rare and unlikely to be responsible for the macropathology observed in almost 5% of the Con Co Ngua assemblage.

The histopathological outcomes of Con Co Ngua are consistent with clinical bone histological observations in cases of beta thalassaemia. Increased osteoclast-mediated resorptive activity, and decreased osteoblastic activity have both been described as underling processes increasing porosity of the endocortical margins of the long bones leading to osteoporosis^23^. Disruptions to bone formation and mineralisation as a result of focalised (secondary) osteomalacia and iron overload, and enhanced osteoclast activation (RANKL factor) in response to medullary expansion, underlie the process of bone metabolism in beta thalassaemia patients^23-25^. The overall histological bone pattern observed in the Con Co Ngua individuals supports localised metabolic changes that were not systemically observed throughout the whole skeleton. Prior histological studies using knockin and knockout mice models have demonstrated variation in the impact on bone microstructure dependent on the mutation responsible for thalassaemia. Some variants emphasised reduction in osteoblastic activity whereas others increased osteoclastic activity^24,26^. Vogiatzi et al.^27^ recorded an overall decreased bone turnover rate. All variants led to decreased bone mineral density of the cortices of the long bones^24,26,27^. Additionally, hormonal co-factors in thalassaemia such as thyroid, parathyroid, estrogen, progesterone, and testosterone dysregulation further increases bone resorption effects^23^. Secondary hypothyroidism is common complication of thalassaemia and may be contributing to the increased cortical thickness, particularly of CCN13M67a, the adolescent who would have been still growing at the time of death ^28^. The endo-cortical porosity of CCN13M59a (old adult) is considerably more advanced than that of CCN13M67a (adolescent) and CCN13M40a (young adult). Age is possibly a factor in the degree of osteoclastic mediated absorption observed in the Con Co Ngua individuals. In conjunction with normal age-related osteoporosis, in beta-thalassaemia hematopoietic autophagy (red blood cell catabolism) is disrupted. This disruption subsequently accelerates bone age and contributes to disordered bone homeostasis^29^.

**References**

1 Williams, H., Davies, A. & Chapman, S. Bone within a Bone. *Clinical Radiology* **59**, 132-144 (2004).

2 Ortner, D. J. *Identification of Pathological Conditions in Human Skeletal Remains*. 2nd edn, (Academic Press, 2003).

3 Assis, S., Santos, A. L. & Roberts, C. A. Evidence of Hypertrophic Osteoarthropathy in Individuals from the Coimbra Skeletal Identified Collection (Portugal). *International Journal of Paleopathology* **1**, 155-163 (2011).

4 Jaffe, H. L. *Metabolic, Degenerative, and Inflammatory Diseases of Bones and Joints*. (Lea and Febiger, 1972).

5 Rizk, N. N. A., Nasr, F. W. & Frayha, R. A. Aseptic Necrosis in Thalassemia Minor. *Arthritis & Rheumatism: Official Journal of the American College of Rheumatology* **20**, 1147-1148 (1977).

6 Lewis, M. Thalassaemia: Its Diagnosis and Interpretation in Past Skeletal Populations. *International Journal of Osteoarchaeology* **22**, 685-693 (2012).

7 Chappard, C. *et al.* 3D Characterization of Pores in the Cortical Bone of Human Femur in the Elderly at Different Locations as Determined by Synchrotron Micro-Computed Tomography Images. *Osteoporosis International* **24**, 1023-1033 (2013).

8 Pitfield, R., Miszkiewicz, J. J. & Mahoney, P. Cortical Histomorphometry of the Human Humerus during Ontogeny. *Calcified Tissue International* **101**, 148-158 (2017).

9 Pfeiffer, S. Cortical Bone Histology in Juveniles. *Documenta Archaeobiologiae* **4**, 15-28 (2006).

10 Lamm, C. *et al.* Micro-CT Analyses of Historical Bone Samples presenting with Osteomyelitis. *Skeletal Radiology* **44**, 1507-1514 (2015).

11 Schultz, M. & Schmidt-Schultz, T. H. Is it Possible to Diagnose TB in Ancient Bone using Microscopy? *Tuberculosis* **95**, S80-S86 (2015).

12 Schultz, M. Paleohistopathology of Bone: A New Approach to the Study of Ancient Diseases. *American Journal of Physical Anthropology* **116**, 106-147 (2001).

13 Snoddy, A. M. E., Buckley, H. R. & Halcrow, S. E. More than Metabolic: Considering the Broader Paleoepidemiological Impact of Vitamin D Deficiency in Bioarchaeology. *American Journal of Physical Anthropology* **160**, 183-196 (2016).

14 Nesterova, A. P. *et al.* *Disease Pathways: An Atlas of Human Disease Signaling Pathways*. (Elsevier, 2019).

15 Maurizi, A. & Teti, A. in *Principles of Bone Biology* Vol. 2 (eds J. P. Bilezikian, T. J. Martin, T. L. Clemens, & C. J. Rosen) 1553-1568 (Elsevier, 2020).

16 Anselmo, D., e Silva, F. C. & Fernandes, T. Characterization of Hypertrophic Osteoarthropathy in an Identified Skeleton from Évora, Portugal, using Combined and Comparative Morphology and Microscopy. *International Journal of Paleopathology* **12**, 11-16 (2016).

17 Von Hunnius, T. Using Microscopy to Improve a Diagnosis: An Isolated Case of Tuberculosis‐Induced Hypertrophic Osteopathy in Archaeological Dog Remains. *International Journal of Osteoarchaeology* **19**, 397-405 (2009).

18 Brickley, M., Ives, R. & Mays, S. *The Bioarchaeology of Metabolic Bone Disease (2nd Edition)*. (Academic Press, 2020).

19 Singer, F. R. & Roodman, G. D. in *Principles of Bone Biology* (eds JP Bilezikian, TL Clemens, TJ Martin, & CJ Rosen) 1601-1613 (Elsevier, 2020).

20 Roches, E., Blondiaux, J., Cotten, A., Chastanet, P. & Flipo, R. M. Microscopic Evidence for Paget's Disease in Two Osteoarchaeological Samples from Early Northern France. *International Journal of Osteoarchaeology* **12**, 229-234 (2002).

21 Wenstrup, R., Roca-Espiau, M., Weinreb, N. & Bembi, B. Skeletal Aspects of Gaucher Disease: A Review. *The British Journal of Radiology* **75**, A2-A12 (2002).

22 Stowens, D. W., Teitelbaum, S. L., Kahn, A. J. & Barranger, J. A. Skeletal Complications of Gaucher Disease. *Medicine* **64**, 310-322 (1985).

23 Perisano, C. *et al.* Physiopathology of Bone Modifications in β-thalassemia. *Anemia* **2012**, 320737 (2012).

24 Thongchote, K. *et al.* Bone Microstructural Defects and Osteopenia in Hemizygous βIVSII-654 Knockin Thalassemic Mice: Sex-Dependent Changes in Bone Density and Osteoclast Function. *American Journal of Physiology-Endocrinology and Metabolism* **309**, E936-E948 (2015).

25 Wong, P., Fuller, P. J., Gillespie, M. T. & Milat, F. Bone Disease in Thalassemia: A Molecular and Clinical Overview. *Endocrine Reviews* **37**, 320-346 (2016).

26 Charoenphandhu, N. *et al.* Responses of Primary Osteoblasts and Osteoclasts from Hemizygous β-Globin Knockout Thalassemic Mice with Elevated Plasma Glucose to 1, 25-dihydroxyvitamin D 3. *Scientific Reports* **9**, 1-14 (2019).

27 Vogiatzi, M. G. *et al.* Changes in Bone Microarchitecture and BiomechanicalPproperties in the th3 Thalassemia Mouse are Associated with Decreased Bone Turnover and Occur during the Period of Bone Accrual. *Calcified Tissue International* **86**, 484-494 (2010).

28 Coindre, J.-M. *et al.* Bone Loss in Hypothyroidism with Hormone Replacement: A Histomorphometric Study. *Archives of Internal Medicine* **146**, 48-53 (1986).

29 Yuan, Y. *et al.* Deterioration of Hematopoietic Autophagy is Linked to Osteoporosis. *Aging Cell*, e13114 (2020).
